# Quality of life in children and adolescents with blood coagulation disorders and hemoglobinopathies

**DOI:** 10.1590/1807-3107bor-2024.vol38.0052

**Published:** 2024-06-24

**Authors:** Leandro Tavares da SILVA, Carolina Mendes FRUSCA-DO-MONTE, Gabriela Silva ALMEIDA, Victor Cordeiro da SILVA, Claudia Santos LORENZATO, Cristiane Baccin BENDO, José Vítor Nogara Borges MENEZES, Cassius Carvalho TORRES-PEREIRA, Fabian Calixto FRAIZ

**Affiliations:** (a) Universidade Federal do Paraná – UFPR, School of Dentistry, Department of Stomatology, Curitiba, PR, Brazil.; (b) Paraná State Center of Hematology and Hemotherapy – HEMEPAR), Curitiba, PR, Brazil.; (c) Universidade Federal de Minas Gerais – UFMG, School of Dentistry, Department of Pediatric Dentistry, Belo Horizonte, MG, Brazil.

**Keywords:** Blood Coagulation Disorders, Hemoglobinopathies, Quality of Life, Child, Adolescent, Oral Health

## Abstract

The aim of this study was to evaluate the impact of oral conditions and health-related quality of life (HRQoL) on oral health-related quality of life (OHRQoL) in children and adolescents with blood coagulation disorders and hemoglobinopathies (BCDH). The study was cross-sectional and included 61 individuals aged 2 to 18 years with BCDH. Exams for dental caries (dmft/DMFT index), oral hygiene (simplified oral hygiene index – OHI-S), and gingival health (modified gingival index – MGI) were performed. The pediatric quality of life inventory™ (PedsQL™) generic core scale and oral health scale were used to measure HRQoL and OHRQoL. Spearman’s correlation coefficient (ρ) and the Mann-Whitney test (α = 0.05) were conducted to assess the relationship between covariates and the PedsQL™ oral health scale. The mean PedsQL™ oral health scale score was 76.66 (SD = 21.36). Worse OHRQoL was correlated with poor oral hygiene (ρ = -0.383; p: 0.004), poor gingival health (ρ = -0.327; p = 0.014), and better HRQoL (ρ = 0.488; p < 0.001). Greater untreated dental caries experience was associated with worse OHRQoL (p = 0.009). Worse oral health status in children and adolescents with BCDH negatively impacts OHRQoL, and OHRQoL and quality of life analyzed from a generic perspective are positively correlated constructs in this population.

## Introduction

Quality of life is a subjective attribute: it is the subject’s perception of their physical and emotional health, their social, economic, spiritual, and occupational relationships, and relies on the individual’s interpretation of what is good or bad.^1^ Thus, quality of life can be interpreted as one’s perception of reality in relation to what one wants or needs. Oral changes can affect the physical and psychosocial health of an individual, for example making it difficult for them to eat, leading to poor oral health-related quality of life (OHRQoL).^2^

Oral health problems can have a strong impact on the OHRQoL of individuals with systemic health conditions^3,4^ as their presence has been reported among those with blood coagulation disorders and hemoglobinopathies (BCDH).^5,6^

The characteristics of each inherited hematological disorder and its oral manifestations may contribute to the lower OHRQoL of these individuals. People with blood coagulation disorders, such as hemophilia and von Willebrand’s disease, have a higher risk of ‘spontaneous’ bleeding, especially in joints, muscles, and soft tissues. This risk is even higher in individuals with the most severe manifestations of the disorder^7^ and can be an important inhibitory factor for oral health self-care. Moreover, the patient’s concern about the bleeding risk in invasive dental procedures may prevent them from seeking dental care.^8^

In hemoglobinopathies, such as sickle cell disease, there is no increased risk for spontaneous bleeding. However, some authors believe that people with sickle cell disease are more susceptible to the development of oral manifestations.^9^

OHRQoL of children and adolescents with hematological disorders, as well as their families, is influenced by the clinical manifestations and the entire social and emotional context associated with these disorders.^5,6^In addition, these individuals live with barriers to accessing dental care, such as professional refusal when there is an increased risk of bleeding.^10^Little is known, however, about which oral condition, and with what intensity, affects OHRQoL of individuals with BCDH.

Although the Brazilian Public Health System (SUS) includes specialized care for this group, a better understanding of the impact of oral conditions on the quality of life of these individuals and their family nucleus can contribute to the improvement of existing care and treatment protocols. This includes seeking constant adaptation to the oral health demands from these groups. Therefore, the aim of this study was to evaluate and understand the impact of oral conditions and HRQoL on OHRQoL in children and adolescents with BCDH.

## Methodology

### Study design and setting

The research project was submitted to and approved by the Research Ethics Committees of the Division of Health Sciences of the Federal University of Paraná – UFPR (CAAE: 12901219.5.0000.0102) and of the Workers’ Hospital of the State Health Department of Paraná – SESA/PR (CAAE: 12901219.5.3001.5225), and followed the STROBE statement.^11^A cross-sectional study was carried out between August 2019 and February 2020 with children and adolescents aged between 2 and 18 years with BCDH, treated at the dental service of the Paraná Center of Hematology and Hemotherapy – Hemepar (Curitiba, Brazil), also including their parents or guardians.

The sample size was estimated through sample calculation for descriptive studies with continuous variables. The values obtained by Bendo et al.^12^ for a population of Brazilian children and adolescents aged 2 to 18 years were used as parameters for the PedsQL™ oral health scale (mean = 83.34; standard deviation = 16.63). In this study, a 95% confidence interval was chosen, with a desired accuracy of 5 points above and 5 points below the mean value (total range of 10 points), requiring a minimum sample of 43 individuals. However, due to the characteristics of the dental service at HEMEPAR and the possibility of not completing the questionnaires, all individuals treated during data collection were invited to participate in the research.

In the study, participants whose parents or legal guardians agreed to their participation in the study and signed the free informed consent form before the consultations were included.

### Socioeconomic and clinical data

We designed a structured questionnaire to collect the socioeconomic and demographic data of the children and adolescents and their families: guardian’s schooling, marital status, guardian’s workplace, child’s sex, and guardian’s perception of the child’s oral health, and race/ethnicity. To obtain the clinical data, the children and adolescents were examined by four trained dentists (kappa ≥ 0.80), following the criteria adopted for each index. The dmft/DMFT (decayed, extracted/missing, and filled teeth) index^13^ was used for dental caries, the simplified oral hygiene index – OHI-S^14^ for oral hygiene, and the modified gingival index – MGI^15^ for gingival health.

The presence of untreated dental caries (UDC) was determined by the c/C component of the dmft/DMFT index. Dental caries experience was considered present in those individuals with a dmft/DMFT index equal to or greater than 1. Gingival health was categorized into ‘absence of inflammation’ (no bleeding = 0), ‘mild inflammation’ (not involving the entire marginal gingiva = 1; involving the entire marginal gingiva = 2), ‘moderate inflammation’ = 3, and ‘severe inflammation’ = 4. Oral hygiene was categorized as ‘good’ (no plaque = 0), ‘regular’ (plaque covering up to 1/3 of the tooth surface), and ‘poor’ (plaque covering more than 1/3, up to 2/3 of the tooth surface = 2; plaque covering more than 2/3 of the tooth surface = 3).

The other variables were divided into guardian’s schooling (‘8 years or less’ or ‘more than 8 years’ of formal education), marital status (‘married/in a common-law marriage’ or ‘others = single, widowed, separated’), guardian’s workplace (‘at home = household, retired’ or ‘away from home = formally employed, civil servant or unregistered worker’), child’s sex (‘female’ or ‘male’), and guardian’s perception of the child’s oral health (’positive = very good, good, regular’ or ‘negative = bad, very bad’).

Race/ethnicity was categorized as ‘white’, ‘brown’, ‘black’, ‘others’ = ‘East Asian’ or ‘indigenous’, or ‘undeclared’ according to IBGE’s (Brazilian Institute of Geography and Statistics) self-declaration of color or race.^16^

Reports of refusal of dental care (guardian’s report of a dentist’s refusal to see the child or adolescent due to their underlying disease) was divided into ‘yes’, when it happened, and ‘no’ when it never happened. Children’s and adolescents’ increased risk of gingival bleeding was classified as ‘yes = severe hemophilia A, moderate hemophilia A, severe hemophilia B, moderate hemophilia B, factor VII deficiency’ or ‘no = mild hemophilia A, mild hemophilia B, type I von Willebrand disease, sickle cell disease, thalassemia, spherocytosis’, and ‘individuals without a defined diagnosis’. The latter group was excluded from the association analyses. The hematological status of the individuals was determined through laboratory tests performed by the specialized medical team at HEMEPAR. We accessed these data through the institution’s electronic medical records. Per capita household income was considered numeric, as were all PedsQL™ scales.

### Health-related quality of life and oral health-related quality of life

Health-related quality of life (HRQoL) was measured using the Pediatric Quality of Life Inventory™ - PedsQL™ generic core scale^17^ and oral health-related quality of life (OHRQoL) using the PedsQL™ oral health scale,^18^ translated into and validated for Brazilian Portuguese.^12,19^ For this study, we obtained a use license from Mapi Research Trust (study/protocol reference 176140).

PedsQL™ is a modular instrument to measure the quality of life of both healthy children and adolescents and those with acute or chronic health conditions. It is divided into age groups (2 to 4, 5 to 7, 8 to 12, and 13 to18 years) and presents two parallel instruments for each age group: one to the parents and one to the child or adolescent, except for children aged 2 to 4 years, in which case the instrument is only applied to the parents.^12,17,18^

The PedsQL™ generic core scale is composed of 23 items divided into four dimensions: physical functioning (eight items), emotional functioning (five items), social functioning (five items), and school functioning (five items).^17^The PedsQL™ oral health scale consisted of five items and only one dimension.^18^

All items in the instruments have five response options, recorded on a scale as follows: 0 (never), 1 (almost never), 2 (sometimes), 3 (often), and 4 (almost always), except for questionnaires aimed at children aged 5 to 7 years, in which case the scale was 0 (never), 2 (sometimes), and 4 (almost always). For this age group, a preparatory test provided with the instrument was applied. A separate page with the three-faced response options was used to help the children understand how they should respond.^18,19^ The response options were then reverse-scored and linearly transformed into a 0-100 scale (0 = 100, 1 = 75, 2 = 50, 3 = 25, and 4 = 0).

All items of the PedsQL™ instruments address the difficulty in participating, performing, or being present in some activity according to the perception of the individual or of their guardian. Thus, the closer the mean value is to 100, the less difficulty the respondent faces in the situations described by the instrument, and the better HRQoL or OHRQoL is. The closer the mean value is to zero, the greater the difficulty and the poorer HRQoL or OHRQoL.

### Data consolidation and statistical analysis

We performed data statistical analysis using the SPSS software (IBM Corp.Released 2017.IBM SPSS Statistics for Windows, Version 25.0. Armonk, NY: IBM Corp.).

The PedsQL™ oral health scale values were not normally distributed (Kolmogorov-Smirnov test, p= 0.022). We used Spearman’s correlation coefficient (ρ) and the Mann-Whitney test to assess the relationship between the covariates and the PedsQL™ oral health scale. To interpret the magnitude of the correlations, we adopted the following classification: (ρ = 0.20 to 0.39 indicated a weak correlation, ρ = 0.40 to 0.69, a moderate correlation, ρ = 0.70 to 0.89, a strong correlation, and ρ > 0.89, a very strong correlation).

To obtain the total values of the PedsQL™ generic core and oral health scales, we summed the self-report values of children and adolescents aged 5 to 18 years (5 to 7, 8 to 12, and 13 to18 years) and the parental proxy-report values for children aged 2 to 4 years.

## Results

During the study,, 69 children and adolescents between the ages of 2 and 18 years were admitted to HEMEPAR’s dental service. Eight guardians did not agree to participate in our study (response rate: 88.4%). Thus, the final sample included 61 children and adolescents and their guardians.

We present the socioeconomic and demographic characteristics of the children and adolescents and their guardians in Table 1. Most of the children and adolescents were male (68.9%), and their mean age was 8.4 years (SD = 4.7). Most of the guardians self-identified as white (59.0%) and 62.7% had attended school for more than 8 years. The median household income per capita was R$ 500.00 (Min: R$100.00; Max: R$ 5,000.00) and with an average of R$ 758.35 (SD = 814.28).

**Table 1:** Socioeconomic and demographic characteristics of children and adolescents and their guardians (Hemepar, Curitiba, Brazil; n = 61).

Variables	n (%)
Sex of the children and adolescents	
Male	42 (68.9)
Female	19 (31.1)
Race/ethnicity of the children and adolescents	
White	36 (59.0)
Brown	9 (14.8)
Black	7 (11.5)
Other	2 (1.8)
Undeclared	7 (11.5)
Race/ethnicity of the guardians	
White	24 (39.3)
Brown	8 (13.1)
Black	7 (11.5)
Other	0 (0.0)
Undeclared	22 (36.1)
Years of education of the guardian*	
≤ 8 years	22 (37.3)
> 8 years	37 (62.7)

*n values lower than 61 represent unfilled items.

Most children and adolescents (69.5%) had dental caries experience (dmft/DMFT) ≥ 1) and 54.1% had at least one tooth with a UDC lesion (C/c component of DMFT or dmft). Only 21.4% of the children and adolescents had no signs of gingivitis. Oral hygiene was considered good in only 12.7% of the children and adolescents. It was not possible to perform the clinical examinations in two children because of difficulty in dental management (Table 2).

**Table 2:** Data from the clinical evaluation of children and adolescents with blood coagulation disorders and hemoglobinopathies (HEMEPAR, Curitiba, Brazil; n = 59*).

Variables	n (%)
Presence of UDC (dmft/DMFT)	
No	28 (45.9)
Yes	33 (54.1)
Gingival health (MGI)	
Absence of inflammation	12 (21.4)
Mild inflammation	38 (67.8)
Moderate inflammation	5 (9.0)
Severe inflammation	1 (1.8)
Oral hygiene (OHI-S)	
Good	7 (12.7)
Regular	42 (77.6)
Poor	6 (10.9)

Two individuals without clinical examination were excluded; *n values lower than 59 represent unfilled items.

Out of the 61 children and adolescents in our study, 30 carried inherited blood coagulation disorders, mainly hemophilia A (n = 21). Twenty-nine children and adolescents had hemoglobinopathies, mainly sickle cell disease (n = 22). Two children/adolescents did not have a defined diagnosis (Table 3).

**Table 3:** Descriptive analysis of groups of underlying diseases and other related aspects (Hemepar, Curitiba, Brazil; n = 61).

Variables	n (%)
Blood coagulation disorders	30 (49.1)
Hemophilia A	21 (34.4)
Hemophilia B	3 (4.9)
Von Willebrand disease	4 (6.5)
Other blood coagulation disorders	2 (3.3)
Inherited hemoglobinopathies	29 (47.9)
Sickle cell disease	22 (36.1)
Thalassemia	4 (6.6)
Other inherited hemoglobinopathies	3 (4.9)
Other unspecified diseases	2 (3.3)
Guardian’s perception of the oral health of the children and adolescents	
Positive	36 (59.0)
Negative	25 (41.0)
Report of refusal of dental care*	
No	49 (84.4)
Yes	9 (15.6)
Increased risk of gingival bleeding due to underlying disease	
No	36 (59.0)
Yes	25 (41.0)
Guardians’ perception of gingival bleeding in the children and adolescents*	
No	42 (70.0)
Yes	18 (30.0)

*n values lower than 61 represent unfilled items.

We observed an increased risk of bleeding due to underlying disease in 41% of the children and adolescents. Guardians reported bleeding gingiva for 30.0% of the children and adolescents. Most of the guardians considered the children’s and adolescents’ oral health to be positive (59.0%) (Table 3).

Table 4 presents the descriptive analysis of the dimensions of PedsQL™ generic core scales, with lower values for emotional aspects (mean: 68.54; SD: 22.71) and school problems (mean: 64.46; SD: 23.30). We obtained a total mean value of 76.66 and a standard deviation of 21.36 in the PedsQL™ oral health scale.

**Table 4:** Descriptive analysis of the Brazilian version of PedsQL™ in its ‘generic core scale 4.0’ and ‘oral health scale 3.0’ (Hemepar, Curitiba, Brazil; n = 61).

Scale	Mean (SD)	Min-Max
Physical functioning	75.61 (21.78)	12.50–100
Emotional functioning	68.54 (22.71)	0–100
Social functioning	78.68 (22.30)	0–100
School functioning	64.46 (23.30)	0–100
Total score: generic core scale	72.64 (19.41)	8.70–100
Total score: oral health scale	76.66 (21.36)	0-100

Table 5 shows a moderate negative correlation between the PedsQL™ oral health scale and the child’s or adolescent’s age (ρ = -0.491; p < 0.001); a weak negative correlation between the PedsQL™ oral health scale oral hygiene (ρ = -0.383; p = 0.004) and gingival health (ρ = -0.327; p = 0.014); and a moderate positive correlation between the PedsQL™ oral health scale and the PedsQL™ generic core scales (ρ = 0.488; p < 0.001).

**Table 5:** Correlation between ‘PedsQL™ oral health scale 3.0’ and other covariates (Hemepar, Curitiba, Brazil; n = 60).

Variables	PedsQL™ oral health scale	p-value	Magnitude of the correlation

	Correlation coefficient*		
Age (n = 60)	-0.491	< 0.001	moderate
Income *per capita* (n = 54)	0.111	0.424	-
Oral hygiene - OHI-S (n= 55)	-0.383	0.004	weak
Gingival health - MGI (n = 56)	-0.327	0.014	weak
PEDSQL™ generic core scale *4.0* (n = 60)	0.488	< 0.001	moderate

*Nonparametric Spearman’s Rho Test; significant p values highlighted in bold.

A greater presence of UDC was associated with worse OHRQoL (lower mean values on the PedsQL™ oral health scale). Guardians’ positive reporting of the oral health and lower perception of gingival bleeding in the children and adolescents were associated with better OHRQoL (higher mean values on the PedsQL™ oral health scale) (Table 6).

**Table 6:** Values of ‘PedsQL™ oral health scale 3.0’ in function of the other covariates (Hemepar, Curitiba, Brazil; n = 60).

Variables (n)	Mean (SD)	Median	Min-Max	p-value*
Sex of the children and adolescents				
Female (19)	76.05 (20.31)	80.00	30.00–100	0.791
Male (41)	76.95 (22.07)	80.00	0–100	
Guardian’s marital status				
Married/in a common law marriage (45)	78.44 (21.18)	80.00	0–100	0.572
Other (13)	74.61 (21.55)	80.00	35.00–100	
Guardian’s years of education				
≤ 8 years (21)	78.33 (19.12)	80.00	35.00–100	0.948
> 8 years (37)	77.16 (22.43)	80.00	0–100	
Presence of untreated dental caries				
Yes (35)	70.28 (22.58)	80.00	0–100	0.005
No (24)	85.00 (16.08)	92.50	50.00–100	
Increased risk of bleeding due to underlying disease				
Yes (23)	75.21 (23.76)	80.00	0–100	0.614
No (33)	79.09 (20.01)	80.00	0–100	
Guardian’s perception of the children’s and adolescents’ oral health				
Positive (36)	83.33 (16.34)	87.50	50.00–100	0.006
Negative (24)	66.66 (24.30)	72.50	0–100	
Guardian’s perception of children’s and adolescents’ gingival bleeding				
Yes (18)	62.50 (15.45)	60.00	35.00–100	< 0.001
No (41)	82.68 (21.03)	85.00	0–100	

*Mann-Whitney Test, significant values highlighted in bold.

## Discussion

Analyzing HRQoL and OHRQoL and the factors that may influence them in children and adolescents with BCDH is fundamental to understand their needs and formulate and apply public health policies. In this study, we observed that dental caries, gingival inflammation, and oral hygiene influenced the OHRQoL of children and adolescents with BCDH. We also observed a correlation between parents’ perception of their children’s oral health and gingival bleeding and the children’s and adolescents’ OHRQoL, as well as a correlation between HRQoL and OHRQoL.

OHRQoL has been studied in several populations of healthy individuals^12,21^ and in those with acute or chronic systemic involvement,^3,4,22^ especially in the last few decades. It has been observed that individuals with systemic conditions present a worse OHRQoL than healthy individuals.^3,4,22^ However, due to the wide variety of clinical manifestations of the various systemic conditions, it is plausible to consider that each condition affects the OHRQoL of carriers and their families in different ways and with different intensity.

Since BCDH have a low prevalence, it is essential to record the findings for various services and populations. However, few studies have assessed OHRQoL in individuals with hematologic changes.^23-25^ Due to the low prevalence of these conditions, the samples are often small in these studies, which makes interpretation and comparison of data more complex. In addition, the wide variety of instruments developed to assess OHRQoL also contributes to this challenge.^26-28^

The impact of oral conditions on the OHRQoL of children and adolescents has been assessed using PedsQL™ oral health scale in healthy individuals^29^ and in those with systemic diseases.^3,4^ The mean values of OHRQoL (from the PedsQL™ oral health scale) in children and adolescents without systemic conditions were higher than those found in our study.^29^ On the other hand, Cardoso et al.^4^ obtained OHRQoL (PedsQL™ oral health scale) values in adolescents with cerebral palsy that were very close to what we found for children and adolescents with BCDH. It is important to highlight the differences between the health problems presented, as individuals with cerebral palsy have different needs from patients with BCDH, such as changes in perception and cognitive deficit.^30^

Among the oral manifestations, dental caries is considered one of the main factors related to lower OHRQoL in children and adolescents, as they can lead to problems such as pain, infection, and systemic complications.^31^ Therefore, it has been shown to be an important factor in the decline of OHRQoL, both in healthy children and adolescents^21,29^and in those with systemic conditions such as cerebral palsy^4^ and chronic kidney disease.^3^ The results of this study confirm the association between dental caries and OHRQoL in children and adolescents with BCDH. This may be related to the fear or anxiety these individuals face due to the risk of bleeding during oral health care,^32^, financial barriers, or lack of dentists trained to treat them.^33^ This specificity can lead to the postponement of relatively simple dental procedures and thus cause the worsening of dental caries and significant pain.

In addition, in this study, gingival conditions and poorer oral hygiene of children and adolescents with BCDH were also correlated with OHRQoL. The literature shows that, in healthy individuals, episodes of gingival bleeding and the presence of periodontal disease^29^ influence OHRQoL. The same relationship has been observed in patients with cerebral palsy^4^ and chronic kidney disease.^3^

It is important to note that in individuals with BCDH, gingival health and oral hygiene have important specificities and are a cause for concern. Poor oral hygiene has been demonstrated in this population,^34^and it is likely that the fear of bleeding during oral hygiene contributes to this outcome.^32^ However, we should also consider that, in some situations, the opposite behavior can occur. Patients who are well oriented and aware that the increased risk of bleeding is associated with the accumulation of dental biofilm and not with the hematological alteration itself can establish an excellent standard of oral hygiene to avoid the accumulation of dental biofilm and, consequently, the bleeding.^35^

In addition to clinical conditions, in this study, parents’ reports of their children’s oral health conditions were associated with children’s and adolescents’ OHRQoL. It is likely that the systemic condition of these individuals and their need for increased health care^36^ will result in more attention to oral health and a greater likelihood that parents will notice oral manifestations. Cardoso et al.,^4^ for instance, observed that a positive perception of oral health was associated with better OHRQoL of individuals with cerebral palsy.

Children and adolescents with hematological alterations may have better oral health conditions, as they are inserted in specialized treatment centers that include health education actions from the first years of life.^32,35^ However, the high prevalence of oral diseases found in this study does not support this statement. It is likely that the differences found are also a result of the type of dental care provided to this population. Services with a great focus on prevention may have a greater positive impact on oral health levels than those more directed to curative treatment. On the other hand, it is suggested that children and adolescents with BCDH may neglect oral hygiene habits due to fear of the possibility of bleeding.^34^

In this study, we found a moderate positive correlation between the PedsQL generic core scale values and the PedsQL™ oral health scale, which confirms the close relationship between general health and oral health.^37^ Differences in the pattern of OHRQoL are expected depending on health conditions. Studies that also used the PedsQL™ generic core scales showed higher OHRQoL values in healthy groups^12^ than studies involving groups with systemic conditions such as cerebral palsy^4^ and chronic kidney disease.^3^

In our study, the social dimension of the PedsQL™ generic core scales was the least compromised. It is suggested that children and adolescents with BCDH have no difficulty socializing with other children and adolescents and participating in activities and games in their friendship circles. On the other hand, the emotional and school dimensions had the lowest mean values. The peculiar routine of a patient with blood disease can lead to challenges in the school environment, mainly due to physical deformities and limitations of functional abilities imposed by their systemic condition.^38^

Studies using questionnaires are subject to memory and information biases, but in the PedsQL™, the items do not refer to earlier than the previous month, which minimizes difficulties related to recalling events. Our study is the first to use the PedsQL™ oral health scale instrument to measure OHRQoL in children and adolescents with BCDH. The PedsQL™ oral health scale is very well suited for studying the OHRQoL of patients with low prevalence diseases, as it can be used over a wide age range with the same conceptual and construction logic. This facilitates the inclusion of many individuals, regardless of age and, consequently, the expansion of the studied group. The use of the PedsQL™ oral health scale to study the OHRQoL in groups with a wide age range, such as research in services aimed at the care of patients with chronic diseases, allows the use of a single instrument that covers the entire life cycle.^18^ Cross-sectional studies – such as ours – are not capable of raising causality; however, they suggest hypotheses that facilitate the design of more appropriate research to define causality.

Even if one of the advantages of the PedsQL™ oral health scale is the possibility of comparing the results of several groups, some precautions are still fundamental, especially regarding the age of the investigated group. Given that the instrument can be applied to children and adolescents aged 2 to 18 years and there is variation in the perception of OHRQoL as a function of age,^17,18^ this aspect should be considered in the comparative analysis of the studies. In addition, the external validity of our study should be considered very carefully.

Further studies are needed to deepen the understanding of how BCDH carriers perceive and behave regarding oral care. In addition, in order to correctly determine which factors are responsible for the observed changes in OHRQoL, studies with designs that have a more appropriate temporal range are needed.

## Conclusion

We conclude that the oral conditions of children and adolescents with BCDH affect their quality of life. In addition, OHRQoL and the quality of life analyzed from a generic perspective are positively correlated constructs in this population.
